# Supplementary material for: Reduced Tolerogenic Program Death-Ligand 1-Expressing Conventional Type 1 Dendritic Cells Are Associated with Rapid Decline in Chronic Obstructive Pulmonary Disease
Source: Cells. 2024 May 20;13(10):878. doi: 10.3390/cells13100878 (PMC11119227; doi:10.3390/cells13100878)
Supplement: Supplementary file 1 [file cells-13-00878-s001.zip › cells-2955639-supplementary.pdf]

Supplementary

Table S1. Characteristics of normal and COPD participants.

	Normal	COPD
Subjects, n	29	54
Sex, n (male/female)	11/18	49/5*
Age (years)	42 ± 11	73 ± 9*
BMI, kg/m²	25 ± 4	23 ± 4
Smoking history		
Never smokers	27	4*
Ex-smokers	0	28*
Current smokers	2	24*
Pack-years (PY)	5.2±21.5	48.7±30.5
Pulmonary function tests		
FVC, L	NA	2.38 ± 0.74
FVC % pred	NA	77.71 ± 19.31
FEV1, L	NA	1.32 ± 0.53
FEV1% pred	NA	55.01 ± 19.38
FEV1/FVC%	NA	54.39 ± 10.39
Spriometric GOLD, n		
I/II/III/IV	NA	6/28/17/3

Data are presented with mean ± standard deviation (SD); BMI: body mass index; COPD: chronic obstructive pulmonary disease; FEV1: forced expiratory volume in 1 s; % pred: % predicted; FVC: forced vital capacity; NA: not available. Differences between groups were determined using the Fisher's exact test and differences between individual variables from two groups were analyzed by the Mann–Whitney U-test. *: $p<0.05$.

Table S2. Antibodies for flow cytometry analysis.

Antigen	Label	Clone	Company
Lineage Cocktail (CD3, CD14, CD19, CD20, CD56)	FITC	UCHT1, HCD14, HIB19, 2H7, HCD56	Biolegend
CD11c	Alexa Fluor® 700	3.9	Thermo Fisher Scientific
CD123	PerCP/Cyanine5.5	6H6	Thermo Fisher Scientific
CD141	PE-Cy7	M80	Biolegend
CD1c	Alexa Fluor® 647	F10/21A3	BD Bioscience
CD4	BV786	SK3	Biolegend
CD8	BV711	RPA-T8	Biolegend
Foxp3	PE-Cy5	PCH101	thermo
HLA-DR	APC/Fire™ 750	L243	Biolegend
Ki-67	BV421	Ki-67	Biolegend
PD-L1	PE	MIH3	Biolegend

Table S3. The comparison of PD-L1+ DCs and its subsets between COPD phenotypes.

	Non-Rapid decliner		Rapid decliner		P value
Subjects, n (%)	36 (80)		9 (20)		
PD-L1 (% of positive DC)	6.71 ± 5.72		2.30 ± 2.23		0.02
PD-L1 (% of positive cDC1)	15.49 ± 18.64		4.61 ± 4.68		0.03
PD-L1 (% of positive cDC2)	12.33 ± 9,53		8.95 ± 10.43		0.27
PD-L1 (% of positive pDC)	22.71 ± 17.90		15.20 ± 10.73		0.32
	GOLD I	GOLD II	GOLD III	GOLD IV	P value

Subjects, n (%)	6 (11.1)	28 (51.9)	17 (31.5)	3 (5.5)	
PD-L1 (% of positive DC)	7.75± 5.49	5.06± 4.21	6.82 ± 6.84	5.05± 3.35	0.25
PD-L1 (% of positive cDC1)	20.85± 27.27	10.64±10.78	12.43±10.45	12.49±4.14	0.01
PD-L1 (% of positive cDC2)	7.69 ± 3.93	12.1± 10.56	15.11±14.36	15.3±2.26	0.25
PD-L1 (% of positive pDC)	27.81±18.46	19.13±18.89	26.19±17.32	22.85±1.49	0.74
	**Non-Frequent exacerbator**		**Frequent exacerbator**		**P value**
Subjects, n (%)	40 (76.9)		12 (23.1)		
PD-L1 (% of positive DC)	5.90 ± 5.44		4.62± 3.78		0.49
PD-L1 (% of positive cDC1)	14.36 ± 18.65		9.91 ± 6.24		0.94
PD-L1 (% of positive cDC2)	12.51 ± 11.97		13.72 ± 10.72		0.57
PD-L1 (% of positive pDC)	21.6. ± 18.88		23.27±15.56		0.66
	**Non-Eosinophilic**		**Eosinophilic**		**P value**
Subjects, n (%)	47 (87.1)		7 (12.9)		
PD-L1 (% of positive DC)	6.18 ± 5.46		4.48±.2.98		0.67
PD-L1 (% of positive cDC1)	12.75 ± 13.57		11.99± 9.48		0.79
PD-L1 (% of positive cDC2)	12.12 ±11.71		6.71 ± 5.72		0.07
PD-L1 (% of positive pDC)	21.29 ± 18.09		31.21±13.81		0.13
	**Mild emphysematous**		**Severe emphysematous**		**P value**
Subjects, n (%)	17 (53.1)		15 (46.9)		
PD-L1 (% of positive DC)	5.34 ± 4.27		6.98 ± 7.267.39		0.71
PD-L1 (% of positive cDC1)	7.39 ± 8.40		9.61 ± 7.78		0.27
PD-L1 (% of positive cDC2)	5.86. ± 4.55		10.46 ± 9.16		0.46
PD-L1 (% of positive pDC)	12.7 ± 14.17		16.63 ± 14.98		0.52

Data are shown as means ± SEM. For each COPD phenotype, Mann-Whitney U test was used to compare the data of the two groups of the phenotype. If there are more than 3 groups of the phenotype, we first use ANOVA analysis followed by Tukey's post hoc tests.
*p< 0.05

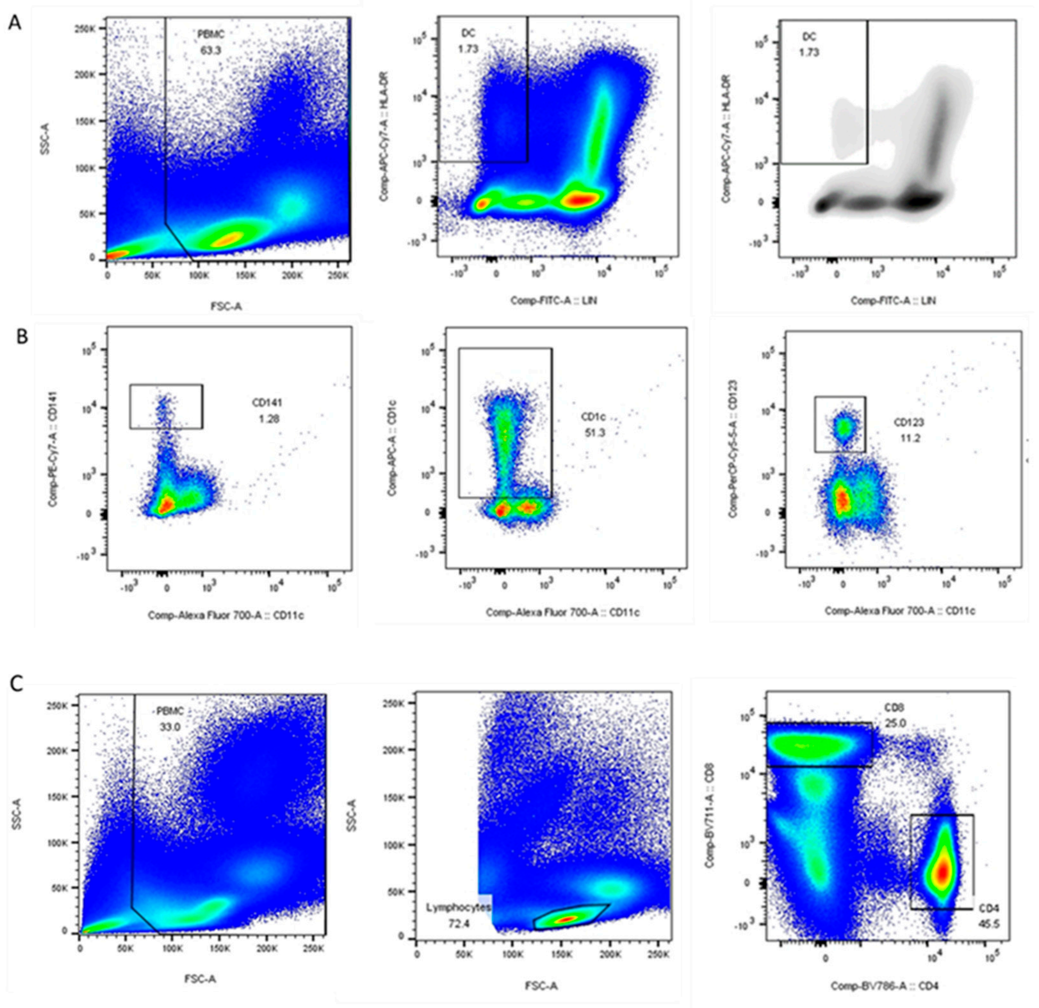

Figure S1. Gating strategies of blood dendritic cells and T lymphocytes. (A) Peripheral blood mononuclear cells (PBMCs) were gated in the FSC/SCC plot (left). Dendritic cells were gated by lineage-negative (lin^-) and HLA-DR-positive ($HLA\text{-}DR^+$) PBMCs (center). Density plots then further confirmed a unique homogeneous group of $Lin\text{-}HLA^-DR^+$ cells (right). **(B)** Among dendritic cells, $CD11c^+CD141^+$ was identified as conventional type 1 dendritic cells (cDC1) (left), $CD11c^+CD1c^+$ was identified as conventional type 2 dendritic cells (cDC2) (middle), and $CD11c^+CD123^+$ was identified are plasmacytoid dendritic cells (pDC) (right). **(C)** Lymphocytes were gated in the FSC/SSC plot (middle). CD4 and CD8 T cells were gated from lymphocytes as shown (right).

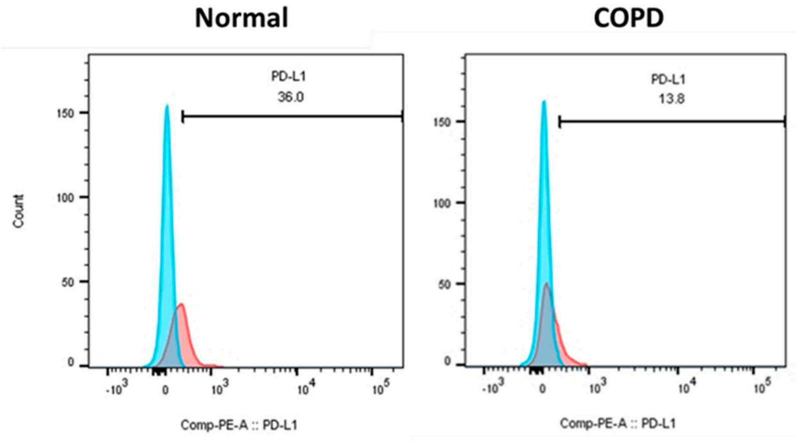

Figure S2. Representative histograms of PD-L1⁺ cDC1 in the blood of normal and COPD participants. Flow cytometric analysis of PD-L1 on cDC1 in PBMCs, representative results from normal and COPD participants. PBMCs were analyzed directly by staining with the indicated antibodies. The percentage of PD-L1⁺ cells was shown in pink. All histograms were gated according to their respective isotype controls (blue).
